# IKBIP promotes tumor development via the akt signaling pathway in esophageal squamous cell carcinoma

**DOI:** 10.1186/s12885-024-12510-4

**Published:** 2024-06-24

**Authors:** Jiannan Hu, Chuanjing Dai, Zhaoji Ding, Yixiao Pan, Lingxiao Lu, Jiaqian Bao, Jingmin Zheng

**Affiliations:** Department of Public Laboratory, Taizhou Hospital of Zhejiang Province Affiliated to Wenzhou Medical University, 150 Ximenjie, Linhai, Zhejiang Province 317000 China

**Keywords:** AKT, ESCC, IKBIP, Prognosis, Tumor-promoting

## Abstract

**Background:**

Esophageal squamous cell carcinoma (ESCC) is one of the most common cancers worldwide. Inhibitor of kappa B kinase interacting protein (IKBIP) has been reported to promote glioma progression, but its role in other cancers remains unclear. This study aimed to investigate the role of IKBIP and its underlying molecular mechanisms in ESCC.

**Methods:**

The mRNA expression of IKBIP was analyzed using multiple cancer databases. Immunohistochemistry was performed to detect IKBIP protein expression in ESCC tissues and adjacent normal tissues, and Kaplan‒Meier survival and Cox regression analyses were carried out. The effects of IKBIP knockdown (or overexpression) on ESCC cells were detected by cell viability, cell migration, flow cytometry and Western blot assays. LY-294002 was used to validate the activation of the AKT signaling pathway by IKBIP. Finally, the role of IKBIP in ESCC was verified in a xenograft model.

**Results:**

Both bioinformatics analysis and immunohistochemistry indicated that IKBIP expression in ESCC tissues was significantly increased and was associated with the prognosis of ESCC patients. In vitro experiments revealed that IKBIP knockdown significantly inhibited the proliferation and migration of ESCC cells, and induced cell apoptosis and G1/S phase arrest. Molecular mechanism results showed that the AKT signaling pathway was further activated after IKBIP overexpression, thereby increasing the proliferation and migration abilities of ESCC cells. In vivo study confirmed that IKBIP promoted the initiation and development of ESCC tumors in mice.

**Conclusions:**

IKBIP plays a tumor-promoting role in ESCC and may serve as a predictive biomarker and a potential therapeutic target for ESCC.

**Supplementary Information:**

The online version contains supplementary material available at 10.1186/s12885-024-12510-4.

## Introduction

Esophageal cancer (ESCA) has become a common malignant tumor worldwide [1]. According to the cancer burden data, the number of ESCA deaths worldwide reached 187,500 in 2022, ranking fifth among malignant tumor deaths [2]. Esophageal squamous cell carcinoma (ESCC) is the main histological subtype of ESCA, accounting for 85.8% of ESCA cases in China [2]. The exact pathogenesis of ESCC remains unclear and may be related to environmental exposure, genetics, unhealthy diets, and other risk factors [3, 4]. Due to the lack of obvious early symptoms of ESCC, more than 35% of patients are already in an advanced stage at the time of diagnosis [5]. In addition, due to the limited efficacy and serious side effects of current treatments for ESCC (including surgical resection, radiotherapy, chemotherapy, and targeted therapy), the five-year survival rate of ESCC patients is still not satisfactory (less than 15%) [6, 7]. To date, there are few biomarkers that can effectively predict ESCC, which is insufficient for identifying potential ESCC populations [8, 9]. Therefore, identifying novel predictive biomarkers and therapeutic targets for ESCC is essential for improving the prognosis of ESCC patients.

Inhibitor of Kappa B kinase-interacting protein (IKBIP), also known as IKIP, was first discovered and studied in 2004 by Hofer-Warbinek et al. [10]. The gene encoding the IKBIP protein is located on human chromosome 12q23.1 and consists of four exons (E1, E2, E3, and E3a), which are alternately spliced to produce three different transcripts (IKBIP-1, IKBIP 2, and IKBIP-3) [10]. A recent study showed that IKBIP can inhibit the activation of nuclear factor kappa B (NF-κB) by inhibiting IKKα/β phosphorylation [11]. Additionally, IKBIP has been reported to maintain the abnormal proliferation of glioblastoma cells by inhibiting the degradation of CDK4 [12]. Importantly, a growing number of studies have shown that IKBIP may serve as a predictive biomarker and a potential therapeutic target for several cancers, such as glioblastoma [13], renal cancer [14] and lung cancer [15]. However, the role of IKBIP in ESCC has not yet been reported. Elucidating the specific role of IKBIP in ESCC and its underlying molecular mechanism will provide valuable information for understanding the pathogenesis of ESCC and facilitating the development of molecular inhibitors targeting ESCC.

The AKT signaling pathway plays an important role in tumorigenesis and provides necessary conditions for the survival of tumor cells by promoting cell cycle progression, inhibiting apoptosis, and improving the drug resistance of tumor cells [16, 17]. Abnormal activation of the AKT signaling pathway is very common in many types of cancer, including ESCC [17]. This activation may be caused by the overexpression of upstream growth factors, mutation of AKT gene, inactivation of PTEN and other carcinogenic factors [18]. Excessive activation of AKT kinase has been reported to increase the activity of multiple cell cycle regulatory proteins [19], such as Cyclin D1, C-myc, p21 and CDK2, thus promoting the cell cycle progression of tumor cells. In addition, the AKT signaling pathway also participates in the process of epithelial–mesenchymal transition (EMT) and plays a key role in tumor metastasis and invasion [20]. In this study, we found that IKBIP expression was significantly increased in ESCC tissues, and was related to the prognosis of ESCC. Overexpression of IKBIP promoted the development of ESCC in vivo and in vitro, which was related to the activation of the AKT signaling pathway by IKBIP. Our results suggested that IKBIP may be a promising therapeutic target for ESCC.

## Materials and methods

### Public database and bioinformatic tools

GEO database (https://www.ncbi.nlm.nih.gov/geo/) was used to obtain GSE199967 (21 pairs of tumor tissues and normal tissues) and GSE164158 (8 pairs of tumor tissues and normal tissues). The datasets downloaded were normalized following standard data processing procedures [21], and the mRNA expression differences of *IKBIP* in tumor tissues and normal tissues were compared by Student’s t-test in R (version 4.3.1, R-Tools Technology, USA). The ESCA datasets of TCGA and GTEx were directly analyzed via the GEPIA 2.0 (http://gepia2.cancer-pku.cn/#index). UALCAN database is a Web portal for in-depth transcriptomic analysis of TCGA samples, and was employed to analyze *IKBIP* methylation differences between tumor tissues and normal tissues, as well as the IKBIP expression differences between different cancer stages and tumor grades [22]. The RNA-seq data of TCGA-ESCA samples were downloaded from GDC database (https://portal.gdc.cancer.gov/) and normal samples were filtered. The tumor samples were were divided into high-expression group and low-expression group according to the median expression of *IKBIP.* Differentially expressed genes (DEGs) were screened according to the criterion of “|fold change| ≥2, FDR value of < 0.05” by the package “limma” in R. Functional annotation and pathway enrichment analysis were carried out by the package “clusterprofile” and visualized by the package “enrichplot” in R [23].

### Clinical samples and patients

A total of 126 ESCC tissue samples and 108 matched normal tissue samples were collected from patients diagnosed with primary ESCC who underwent surgical resection at Taizhou Hospital of Wenzhou Medical University between 2004 and 2018. The 126 ESCC patients included 75 males and 51 females, with a median age of 63 years (ranging from 42 to 82 years) at the time of surgery. None of the included patients received any radiotherapy, chemotherapy or targeted therapy before surgery, and all patients had no previous history of malignancy. Informed consent was obtained from all study subjects. This study was approved by the Ethics Committee of Taizhou Hospital of Zhejiang Province (No: K20210618).

### Immunohistochemistry (IHC) assay

The IHC steps were the same as previously reported [24]. In this study, ESCC tissue and adjacent normal tissue samples were incubated with an anti-IKBIP antibody (#PA5-66401, 1:500, Thermo Scientific, USA). Subsequent staining was carried out according to the instructions of a horseradish peroxidase (HRP)-labeled secondary antibody kit (#cw2069s, CWBIO, Beijing, China). IHC scoring was performed by two experienced pathologists in a blinded manner. The IHC score of each sample was judged according to the staining intensity of the cells (0 for negative, 1 for weak, 2 for moderate, and 3 for strong) and the percentage of positive cells in the tissue (0 for < 10%, 1 for 10–40%, 2 for 41–75%, and 3 for > 76%). The final score of each sample was calculated by adding the staining intensity score and staining degree score. An IHC score of 0–4 was considered low IKBIP expression, and a score of 4 or more was considered high IKBIP expression.

### Cell culture

Human ESCC cell lines KYSE-30, KYSE-150, KYSE-410 and TE-1, normal esophageal epithelial cell line HECC and human embryonic kidney cell line HEK-293T were purchased from the Cell Bank of the Chinese Academy of Science (Shanghai, China). KYSE-30, KYSE-150, KYSE-410, TE-1 and HECC cells were cultured in RPMI-1640 medium (#BC-M-017-500, Biochannel, Liaoning, China) supplemented with 10% fetal bovine serum (FBS) (#BC-SE-FBS01, Biochannel, Jiangsu, China). HEK-293T cells were cultured in high-glucose DMEM (#MA0212, Mellen Biologicals, Jiangsu, China) supplemented with 15% FBS. The cells were cultured in an incubator at 37 ℃ with 5% CO_2_. No antibiotics were added during cell culture. The PI3K/AKT signaling-specific inhibitor LY294002 was purchased from MedChemExpress (#HY-10,108, Monmouth Junction, NJ, USA), and its working concentration in culture medium was 10 µg/mL.

### Lentivirus production and infection

Lentivirus packaging procedures were performed as previously reported [25]. Here, two short hairpin RNAs (sh; sh-1 sequence: 5’-GCTACATCATCCATGTCTTTG-3’; sh-2 sequence: 5’-GGAAGTATCCAACCTCCAAGA-3’) targeting IKBIP were separately inserted into the shuttle plasmid pLVshRNA-EGFP (2 A) to produce the IKBIP-knockdown lentiviruses pLV-IKBIP-sh1 and pLV-IKBIP-sh2. A nonsense sequence was inserted into the backbone of the control lentivirus pLV-NC. In addition, the coding sequence of the IKBIP protein (or an equal length of nonsense sequence) was cloned and inserted into another shuttle plasmid, pCDH-CMV-MCS-EF, to produce the IKBIP-overexpressing lentivirus pCDH-IKBIP (or control lentivirus pCDH-Vector). Subsequently, the above lentivirus was used to infect stable IKBIP-silenced or IKBIP-overexpressing ESCC cells.

### Cell viability assay

Cell viability was determined using CCK-8 reagent (#C0121, Biosharp, Anhui). According to the reagent instructions, 3 × 10^3^ ESCC cells were cultured in 96-well plates. After incubation for a period of time, 10 µL of CCK-8 reagent was added to each well, and the plates were incubated at 37 °C for 2 h. The optical density (OD) of each well was detected at 450 nm using a microplate reader (Thermo Scientific, USA).

### Colony forming assay

A total of 1 × 10^3^ ESCC cells were seeded into 35 mm dishes supplemented with 2 mL of culture medium containing 20% FBS. After 14 days of culture, the cells were fixed, washed with PBS and stained with 0.1% crystal violet solution (#C0121, Beyotime, Beijing, China). The clone clusters in the dishes were scanned and counted by using ImageJ software (version 1.0, NIH, USA).

### Cell migration assay

Cell migration was measured using a transwell chamber (#3422, Corning, NY, USA). Initially, 3 × 10^4^ ESCC cells were suspended in 100 µL of culture medium without FBS. Then, the cell suspension was added to the upper chamber, and 600 µL of culture medium containing 20% FBS was added to the lower chamber. After 48 h of incubation, the cells that passed through the chamber were fixed and stained with 0.1% crystal violet solution.

### Flow cytometry

EdU staining was carried out according to the instructions of the EdU cell proliferation kit (#C0039, Beyotime, Beijing). Briefly, 3 × 10^5^ ESCC cells were cultured in 6-well plates for 24 h. Next, the cells were fixed with 4% paraformaldehyde solution and stained with EdU reagent. The percentage of EdU-positive cells in the cell population was determined by flow cytometry (Beckman, CA, USA).

According to the instructions of the cell cycle assay kit (#C1052, Beyotime, Beijing, China), 5 × 10^5^ nonconfluent ESCC cells were fixed with 70% ethanol, washed with PBS, and stained with propidium iodide (PI). The results of the cell cycle distribution were directly exported by CytExpert software (version 20.1, Beckman, USA).

For cell apoptosis, an Annexin V-APC/7-AAD kit (#CK-A218, Elabscience, Wuhan, China) was used to detect changes in cell apoptosis after IKBIP knockdown, and the clustering results of apoptotic cells were displayed using FlowJo software (version 10.8, BD Biosciences, CA, USA).

### Western blot

Western blot assay was performed as usual [4]. Briefly, ESCC cells were lysed in RIPA buffer (#P0013E, Beyotime, Beijing, China) supplemented with 1% protease inhibitor (#P1005, Beyotime, Beijing, China) at 4℃. The concentration of the isolated protein was determined using a BCA Protein Quantification Kit (#P0009, Beyotime, Beijing, China). The primary antibodies used for immunoblotting were diluted 1:1000 and included antibodies against GAPDH (#P218, CST, USA), IKBIP (#PA5-66401, Thermo Fisher, USA), AKT (#4685, CST, USA), p-AKT (#4060, CST, USA), PI3K (#4257, CST, USA), C-myc (#GTX103436, GeneTex, China), Cyclin D1 (#2978, CST, USA), CDK2 (CY5020, Abways Technology, China), CDK4 (CY5836, 1:1000, Abways Technology, China), E-cadherin (#GTX100443, GeneTex, China), Vimentin (#5741, CST, USA) and MMP2 (#13667, CST, USA). The secondary antibodies used were HRP-conjugated goat anti-mouse antibody (#RS0001, 1:10000, Immunoway, USA) and HRP-conjugated goat anti-rabbit antibody (#RS0002, 1:10000, Immunoway, USA). The gray value of the protein blot was measured using ImageJ software.

### Reverse transcription quantitative PCR (RT‒qPCR)

Total RNA was isolated using TRIzol® reagent (#9109, Thermo Scientific, Japan). Next, the isolated RNA was synthesized into cDNA using PrimeScript™ RT Master Mix reagent (#RR036A, TakaRa, Japan). After that, quantitative PCR (qPCR) was performed according to the manufacturer’s instructions for the TB Green® Premix reagent (#RR820A, TaKaRa, Japan). The primers used for the qPCR assay were IKBIP (forward sequence: 5’-ACCAATACCAGTTACTGAAA-3’; reverse sequence: 5’-CTCAAACTGGGTCATCAAAGA-3’) and GAPDH (forward sequence: 5’-GATGCTGGCGCTGAGTACGT-3’; reverse sequence: 5’-TCTCATGGTTCACACCCATGA-3’). The relative mRNA expression of each detected gene was calculated using the 2^(-ΔΔCt) method.

### Animal experiment

Before the start of animal study, BALB/C nude mice (male, 4-weeks-old) from the Shanghai SLAC Laboratory Animal Co., Ltd (Shanghai, China) were housed in specific pathogen-free environments and received human care for 7 days to acclimatize. Afterwards, the twenty BALB/C nude mice were randomly and equally divided into four groups: the IKBIP-silenced control group (NC), the IKBIP-silenced group (sh-1), the IKBIP-overexpressing control group (Vector) and the IKBIP-overexpressing group (OE). For each mouse, 1 × 10^6^ cells (150-NC (KYSE-150 cells infected with the lentivirus pLV-NC), 150-sh-1 (KYSE-150 cells infected with the lentivirus pLV-IKBIP-sh-1), 150-Vector (KYSE-150 cells infected with the lentivirus pCDH-Vector) and 150-OE (KYSE-150 cells infected with the lentivirus pCDH-IKBIP)) suspended in 0.1 mL of PBS were subcutaneously injected into the side of the armpit. After the injection of tumor cells, the tumor size, body weight and health status of the mice were continuously monitored. The tumor volume (tumor length and width) was measured using a Vernier caliper every 4 days and was calculated according to the formula “1/2 × tumor length × tumor width^2^”. After 24 days of monitoring, all mice were sacrificed by CO_2_ euthanasia, and tumor nodules were isolated and weighed. The isolated tumor tissues were fixed in 4% paraformaldehyde solution, and the expression of Ki-67 protein in tumor tissues was detected by IHC using an anti-Ki-67 antibody (#790–4286, 1:200, Roche, USA). The animal experiment protocol was approved by the Animal Care Committee of Taizhou Hospital of Zhejiang Province (No. ty-2022129).

### Statistical analysis

Statistical analyses and plotting were performed in GraphPad Prism (version 9.0, USA). Differences in overall survival time between groups were compared using the log-rank test. The chi-square test was used to analyze the associations between IKBIP expression and the clinicopathologic parameters of patients. Cox regression analysis was used to determine risk factors related to the prognosis of ESCC patients. Comparisons between two groups were carried out by using student’s t-test. One-way ANOVA was used to compare the differences between multiple groups. Data were presented as mean ± SD (Standard Deviation) from 3 independent assays. Statistical significance was defined as *p* < 0.05.

## Results

### IKBIP expression was upregulated in ESCC tissues and correlated with the prognosis of ESCC patients

**Fig. 1:**
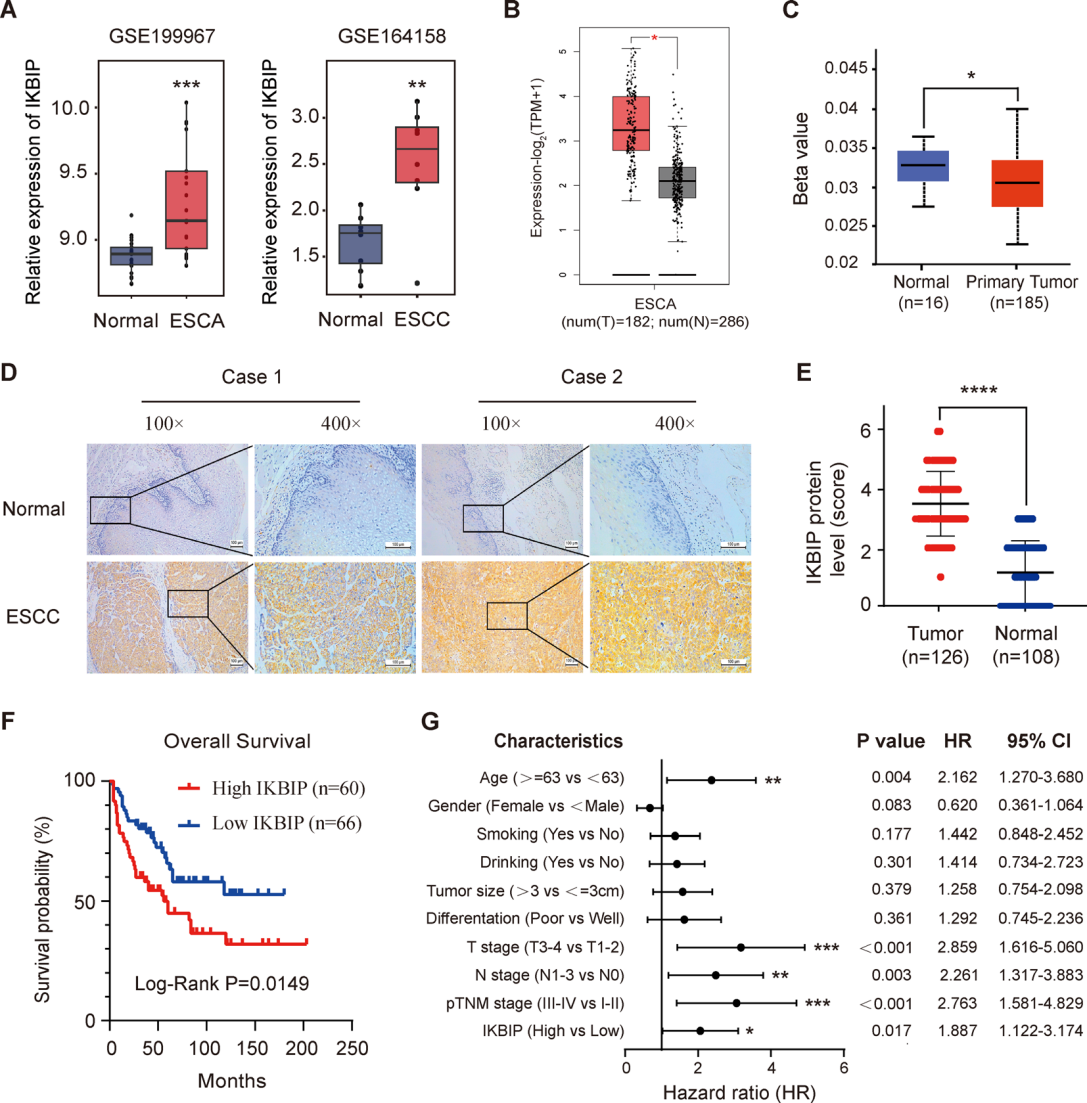
Expression of IKBIP in ESCC tissues and its association with prognosis

To determine the expression of IKBIP in ESCC, we downloaded two GSE datasets from the GEO database, which contain expression profile information from ESCA (GSE199967) and ESCC (GSE164158) tissues. After normalizing the raw expression matrices, we performed differential expression analysis of the *IKBIP* gene in tumor tissues and paired normal tissues and found that *IKBIP* expression was significantly upregulated in both ESCC (*p* < 0.001) and ESCA (*p* < 0.01) tissues (Fig. 1A). Next, we used the GEPIA2 platform to validate the expression of *IKBIP* in ESCA samples from the TCGA database, and the results showed that *IKBIP* was also highly expressed in TCGA-ESCA tissues (*p* < 0.01) (Fig. 1B). Abnormal DNA methylation can reportedly lead to imbalanced gene expression, thereby increasing the risk of tumorigenesis. Therefore, we compared the DNA methylation levels of *IKBIP* in ESCA tissues and normal tissues through the UALCAN database (*p* < 0.05) (Fig. 1C). The results showed that the *IKBIP* gene was hypomethylated in ESCA tissues, which may lead to the upregulation of *IKBIP* expression in tumor tissues.

Furthermore, we detected the expression of the IKBIP protein in 126 ESCC tumor tissues and 108 matched adjacent normal tissues through IHC staining assay. Representative IHC images of 2 patients with ESCC are shown in Fig. 1D. Compared with that in adjacent esophageal tissues, the staining intensity of IKBIP in tumor cells in ESCC tissue was darker, and the cytoplasm of tumor cells was yellow or brown. The boundaries between the cancer tissue and stroma were clear, and the interstitial fibrous tissue was infiltrated by inflammatory cells. The number of positive cells under the microscope was greater than 70% (Fig. 1D). Moreover, we compared the differences in the expression of IKBIP between ESCC tumor tissue and adjacent normal tissue (determined by scoring) using a t test, and the results showed that the expression of IKBIP in ESCC tumor tissue was significantly greater than that in corresponding adjacent tissue (*p* < 0.0001) (Fig. 1E). In addition, we collected the prognostic information of the 126 ESCC patients and divided these patients into high-expression and low-expression groups according to the criteria of the IHC score for IKBIP expression. The relationship between IKBIP expression and overall survival time in ESCC patients is shown in Fig. 1F. Kaplan-Meier survival analysis revealed that ESCC patients with low IKBIP expression had a better prognosis than did those with high IKBIP expression (log-rank test, *p* = 0.019). Furthermore, Cox regression analysis indicated that age (*p* = 0.004), IKBIP expression (*p* = 0.017), T stage (*p* < 0.001), N stage (*p* = 0.003) and TNM stage (*p* < 0.001) were independent indicators for predicting the prognosis of ESCC patients (Fig. 1G).

(A) Comparison of the mRNA expression levels of IKBIP in two datasets (GSE199967 for ESCA samples and GSE164158 for ESCC samples). (B) GEPIA2 database based on TCGA-ESCA samples showed the mRNA expression of IKBIP was higher in tumor tissues than normal tissues. (C) The methylation differences of *IKBIP* in normal tissues and ESCA tissues were compared through the UALCAN database. (D) Representative IHC images of 2 samples of ESCC tumor tissues and their matched nontumor tissues. The IKBIP protein was stained brown and was mainly located in the cytoplasm of ESCC tumor cells; the nuclei were stained blue (magnification: 100× and 400×). Scale bar, 100 μm. (E) Analysis of differential expression of IKBIP in 126 ESCC tissues and 106 adjacent nontumor tissues detected by IHC. (F) Kaplan-Meier survival curve showed the relationship between IKBIP expression and OS in ESCC patients. (G) Cox regression analysis was used to identify risk factors affecting ESCC patient survival. CI: confidence interval. *p < 0.05, **p < 0.01, ***p < 0.001, ****p < 0.0001.

### IKBIP expression was closely associated with clinicopathologic parameters in ESCC patients

**Fig. 2:**
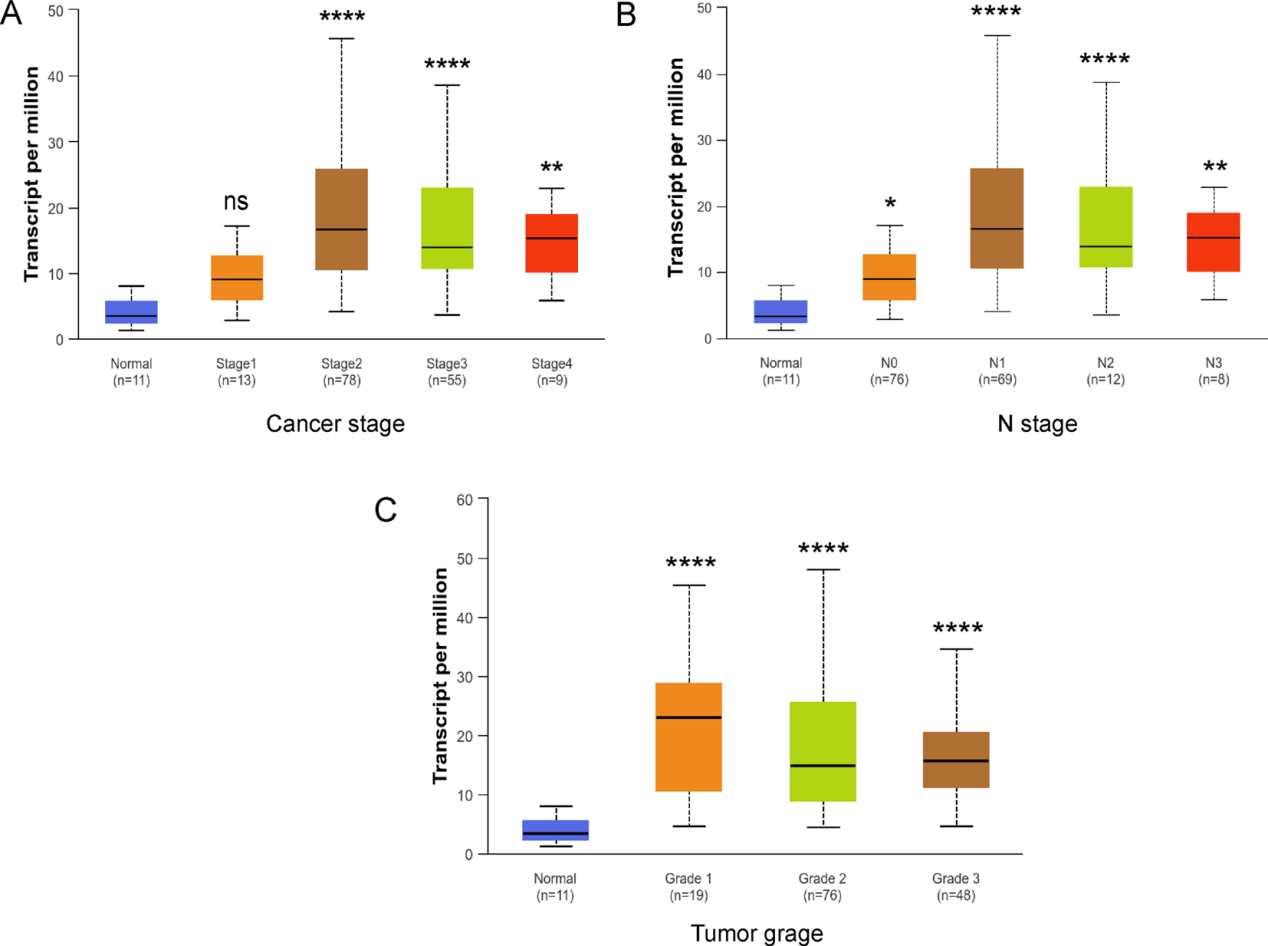
Expression of IKBIP in ESCA based on clinical stage, N stage and tumor grade

To determine the clinical significance of IKBIP expression in ESCC, we collected clinicopathologic parameters of 126 patients with ESCC and divided them into an IKBIP high-expression group and an IKBIP low-expression group according to the IHC score. By analyzing the relationships between IKBIP expression and different clinicopathologic parameters, such as age, sex, tumor differentiation status and TNM stage, we found that tumor length (*p* = 0.028), N stage (*p* < 0.001), and TNM stage (*p* < 0.001) were significantly associated with IKBIP expression (Table 1). Additionally, we evaluated the expression of *IKBIP* in patients with different cancer stages (stage 1–4), N stages (stage N0-N3) and tumor differentiation grades (stage 1–3) through the UALCAN database. The results showed that IKBIP expression was closely correlated with cancer stage (Fig. 2A), N stage (Fig. 2B) and tumor differentiation grade (Fig. 2C), which was consistent with the results of the IHC-based clinicopathological analysis. Based on these results, we demonstrated that IKBIP expression was significantly upregulated in ESCC tissues and was closely related to the clinical stage, tumor stage and overall survival of patients with ESCC. IKBIP may be a predictive biomarker for ESCC.

**Table 1:** Relationships between IKBIP expression and clinicopathologic parameters in ESCC patients

Clinicopathologic parameters	Total	IKBIP (Detected by IHC)		*P* value
		Low group	High group	
Number of cases	126	66	60	
**Age (year)**				0.610
<63	60	30	30	
≥ 63	66	36	30	
**Gender**				0.917
Male	75	39	36	
Female	51	27	24	
**Smoking**				0.431
Yes	55	34	21	
No	71	37	34	
**Drinking**				0.114
Yes	36	23	13	
No	89	43	46	
**Tumor Length(cm)**				**0.028**
≤ 3	54	35	19	
>3	67	30	37	
**Differentiation**				0.890
Well and Moderate	96	50	46	
Poor	28	15	13	
**T stage**				0.058
T1-2	53	33	20	
T3-4	73	33	40	
**N stage**				**<0.001**
N0	61	44	17	
N1-3	62	20	42	
**TNM stage**				**<0.001**
I-II	60	45	15	
III-IV	60	17	43	

A. The UALCAN database showed the expression of *IKBIP* in different individual cancer stages (stages 1–4) in ESCA. B and C. The expression of *IKBIP* at different nodal metastasis stages (N0-N3) and tumor differentiation stages (grades 1–3) in ESCA is shown. *: *p* < 0.05, **: *p* < 0.01, ****: *p* < 0.0001, ns: *p* > 0.05.

### Knockdown of IKBIP inhibited ESCC cell proliferation and colony formation

**Fig. 3:**
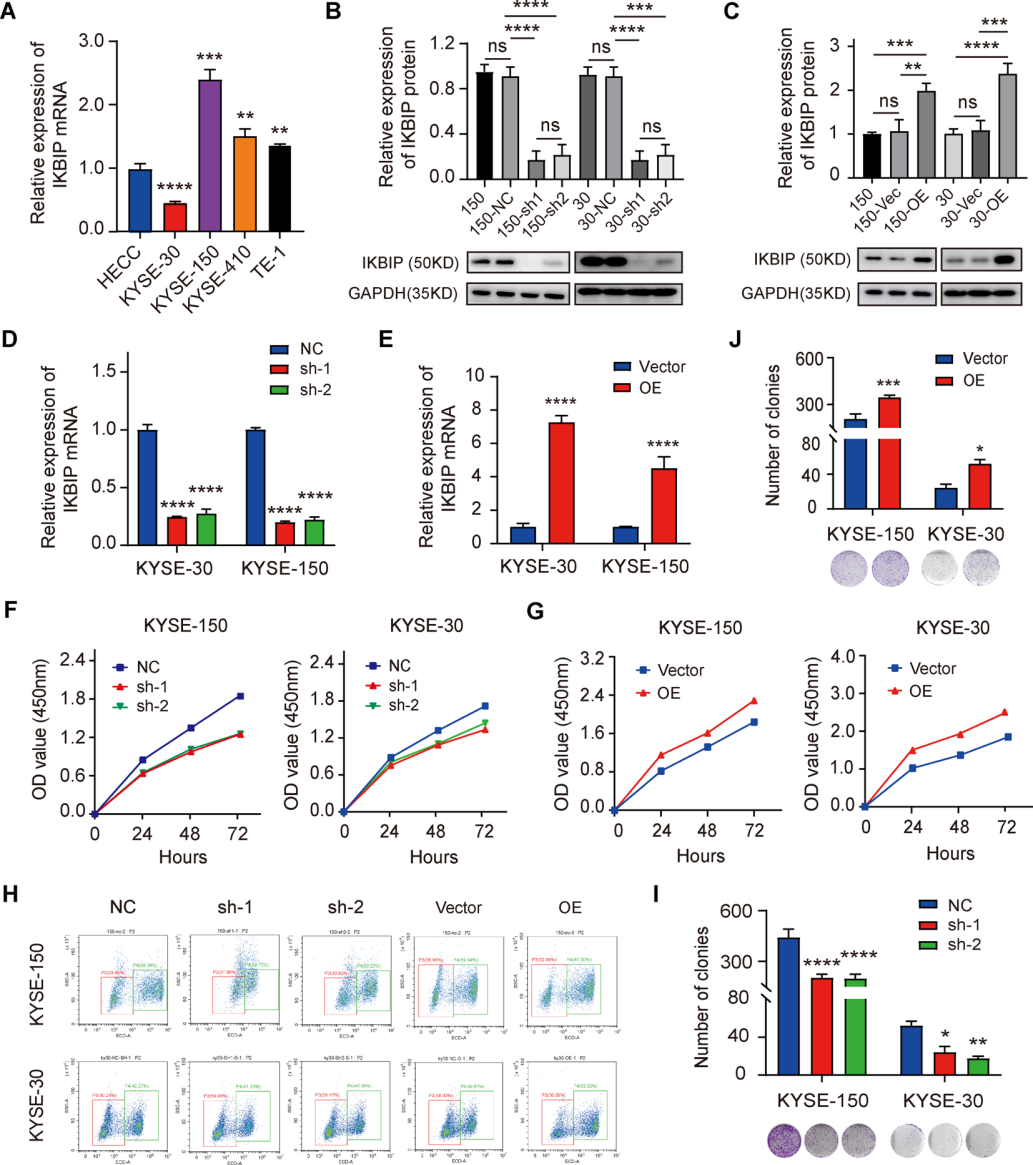
Effects of IKBIP knockdown on ESCC cell proliferation and colony formation

To investigate the specific function of IKBIP in ESCC, we used the human normal epithelial cell line HECC as a control and detected the basic mRNA expression of *IKBIP* in several ESCC cell lines (KYSE-30, KYSE-410, KYSE-150, and TE-1). The RT‒qPCR results showed that the mRNA expression of *IKBIP* in the KYSE-150, KYSE-410 and TE-1 ESCC cell lines was upregulated but was downregulated only in the KYSE-30 cell line (Fig. 3A). Therefore, we selected KYSE-150 cells with naturally high expression of IKBIP and KYSE-30 cells with naturally low expression of IKBIP for subsequent functional experiments. Next, we silenced or overexpressed IKBIP in KYSE-150 and KYSE-30 cells via lentiviral vectors. Western blot analysis revealed that the IKBIP protein expression in 150-sh1, 150-sh-2, 30-sh1 and 30-sh2 cells was markedly downregulated compared with that in their matched control cells (Fig. 3B). In contrast, IKBIP protein expression was significantly increased in 150-OE and 30-OE cells (Fig. 3C). In addition, we validated the expression of *IKBIP* mRNA in IKBIP-silencing (Fig. 3D**)** or IKBIP-overexpressing cells (Fig. 3E**)** by RT‒qPCR, and the results were consistent with the Western blot results. The CCK-8 cell proliferation assay results showed that IKBIP knockdown significantly impaired the proliferation ability of KYSE-150 and KYSE-30 cells (Fig. 3F), while IKBIP overexpression significantly increased the viability of the two ESCC cell lines (Fig. 3G). In addition, we performed an EdU incorporation assay to confirm the effect of IKBIP on ESCC cell proliferation. The flow cytometry results showed that the proportion of ESCC cells that underwent replication and proliferation after IKBIP overexpression was significantly greater than that of control cells without IKBIP overexpression (Fig. 3H). The colony formation assay showed that the colony formation ability of ESCC cells was significantly inhibited after IKBIP knockdown (Fig. 3I), while overexpression of IKBIP enhanced the colony formation ability of ESCC cells (Fig. 3J).

A. The mRNA expression of *IKBIP* in ESCC cell lines (KYSE-30, KYSE-150, KYSE-410 and TE-1) and the human esophageal epithelial cell line HECC was detected by RT‒qPCR. B and C. IKBIP protein expression was detected in ESCC cells with IKBIP knockdown (B) and IKBIP overexpression (C). In this study, the gray value of the protein blot was quantified by ImageJ software, and the GAPDH protein was used as an internal reference for normalization. 150: KYSE-150 cells; 30: KYSE-30 cells. Vec: Vector. D and E. The silencing (D) and overexpression (E) efficiency of IKBIP in ESCC cells was detected by RT‒qPCR. F and G. The viability of ESCC cells with IKBIP inhibition (F) or overexpression (G) for 24, 48 and 72 h was measured through a CCK-8 assay. H. The EDU assay was used to detect changes in the proliferative capacity of ESCC cells after altered IKBIP expression. The clustering results of EDU-positive and EDU-negative cells were directly output from CytExpert software. I and J. The effects of IKBIP knockdown (I) and overexpression (J) on the colony formation ability of ESCC cells were detected, respectively. NC, sh-1 or sh-2: KYSE-150 and KYSE-30 cells infected with the pLV-NC (NC), pLV-IKBIP-sh-1 (sh-1) or pLV-IKBIP-sh-2 (sh-2) lentiviruses. Vector or OE: KYSE-150 and KYSE-30 cells infected with the pCDH-Vector (Vector) or pCDH-IKBIP (OE) lentivirus. *p < 0.05, **p < 0.01, ***p < 0.001, ****p < 0.0001. ns: *p* > 0.05.

### Knockdown of IKBIP inhibited cell migration and induced cell apoptosis and G1/S phase arrest in ESCC cells

**Fig. 4:**
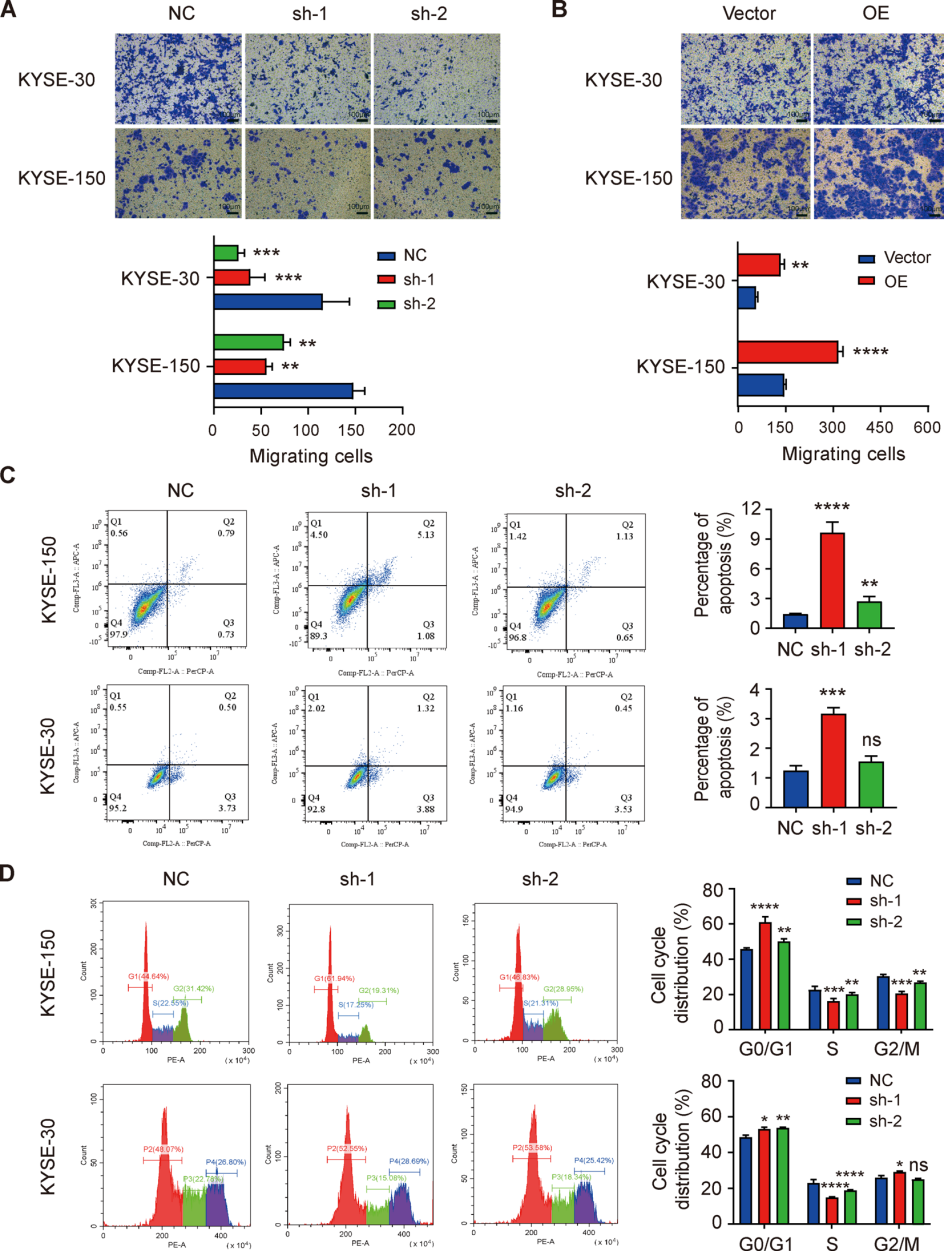
Effects of IKBIP knockdown on cell migration, cell apoptosis and cell cycle distribution

Next, we evaluated the effect of IKBIP knockdown on the migration ability of ESCC cells. We found that compared with that of control cells, the migration ability of KYSE-150 and KYSE-30 cells with low IKBIP expression was significantly weakened (Fig. 4A), while overexpression of IKBIP enhanced the migration ability of the two ESCC cell lines (Fig. 4B). To explore how IKBIP regulates ESCC cell growth, we examined the occurrence of apoptosis after IKBIP knockdown. According to the flow cytometry results, the total percentage of apoptotic cells in the IKBIP-deficient group was significantly greater than that in the control group for both KYSE-150 and KYSE-30 cells (Fig. 4C). Furthermore, we detected the effect of IKBIP knockdown on the cell cycle progression of ESCC cells (Fig. 4D). Analysis of the cell cycle distribution showed that after IKBIP knockdown, the proportion of ESCC cells in the G1 phase increased significantly, while the proportion of ESCC cells in the S phase decreased markedly, which resulted in G1/S phase arrest and inhibited the proliferation of ESCC cells.

A and B. The cell migration assay using transwell chambers showed changes in the migration ability of ESCC cells following IKBIP knockdown (A) or overexpression (B). Scale bar, 100 μm. C. The process of apoptosis was detected by flow cytometry. The clustering results of apoptotic cells are shown on the left, and the statistical results of the quantitative analysis are shown on the right. D. Detection of cell cycle distribution in KYSE-30 and KYSE-150 cells after IKBIP knockdown. NC, sh-1 or sh-2: KYSE-150 and KYSE-30 cells infected with the pLV-NC (NC), pLV-IKBIP-sh-1 (sh-1) or pLV-IKBIP-sh-2 (sh-2) lentiviruses. Vector or OE: KYSE-150 and KYSE-30 cells infected with the pCDH-Vector (Vector) or pCDH-IKBIP (OE) lentivirus. *p < 0.05, **p < 0.01, ***p < 0.001, ****p < 0.0001. ns: *p* > 0.05.

### IKBIP overexpression activated the AKT signaling pathway in ESCC cells

**Fig. 5:**
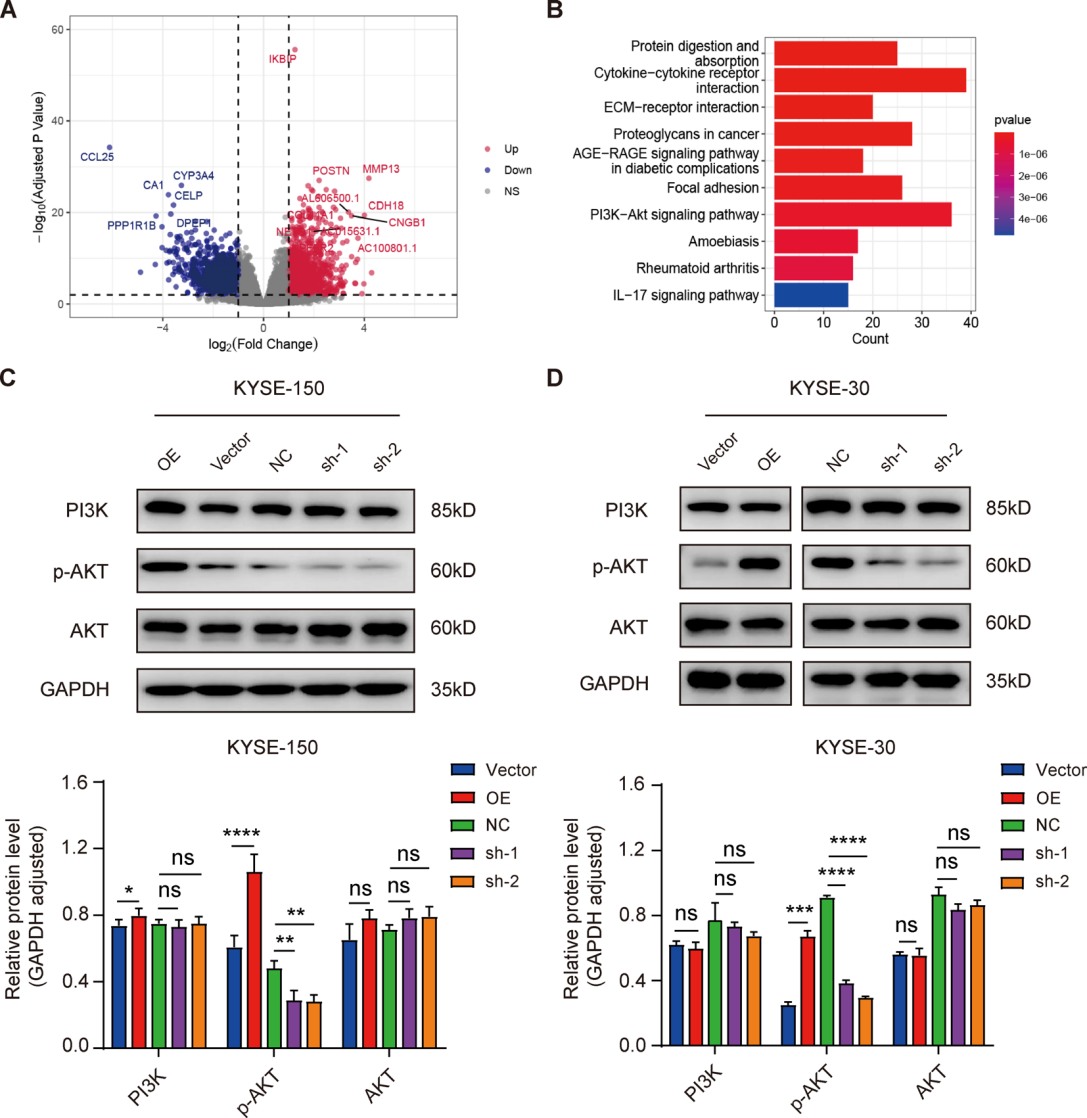
IKBIP participated in the regulation of the AKT signaling pathway in ESCC cells

Based on the above results, we found that IKBIP may play a tumor-promoting role in the process of ESCC. To reveal the potential molecular mechanism by which IKBIP promotes ESCC development, we downloaded RNA-seq data for ESCA patients from the TCGA database. Then, a total of 370 samples from patients who met the criteria for “ESCC” or “primary solid tumor” status were screened and categorized into high-expression and low-expression groups according to IKBIP expression. Based on the screening criterion (|fold change| ≥2, FDR value < 0.05), we identified 3379 DEGs, including 1915 upregulated genes and 1464 downregulated genes (Fig. 5A). Next, we performed GO and KEGG cluster analyses on these upregulated genes. The results showed that among the tumor-related pathways, the PI3K/AKT signaling pathway was clustered, indicating that IKBIP was likely involved in the activation of this signaling pathway (Fig. 5B). To validate the KEGG enrichment results, we detected the expression of proteins related to the PI3K/AKT signaling pathway through Western blotting. The overexpression of IKBIP significantly increased the protein expression of phosphorylated AKT (p-AKT) but had no significant effect on the total protein expression of PI3K or AKT. In contrast, IKBIP silencing reduced the expression of p-AKT in KYSE-150 cells (Fig. 5C). In addition, we also verified the expression of PI3K, AKT and p-AKT in KYSE-30 cells, and the results were consistent with their expression in KYSE-150 cells (Fig. 5D). The above results suggested that IKBIP may be an upstream factor regulating the AKT signaling pathway in ESCC.

(A) According to the criteria (|fold change|) ≥ 2 and FDR value < 0.05, a total of 3379 DEGs were identified in 370 primary ESCC tumor samples, among which the significantly upregulated genes and downregulated genes are shown in the volcano plot. (B) The results of KEGG cluster analysis of upregulated DEGs are shown. C and D. Changes in the expression of PI3K/AKT pathway-related proteins in KYSE-150 (C) and KYSE-30 cells (D) were detected by Western blotting after IKBIP overexpression or knockdown. NC, sh-1 or sh-2: KYSE-150 and KYSE-30 cells infected with the pLV-NC (NC), pLV-IKBIP-sh-1 (sh-1) or pLV-IKBIP-sh-2 (sh-2) lentiviruses. Vector or OE: KYSE-150 and KYSE-30 cells infected with the pCDH-Vector (Vector) or pCDH-IKBIP (OE) lentivirus. *p < 0.05, **p < 0.01, ***p < 0.001, ****p < 0.0001. ns: *p* > 0.05.

### IKBIP overexpression increased the expression of cell proliferation and migration-related proteins in ESCC cells

**Fig. 6:**
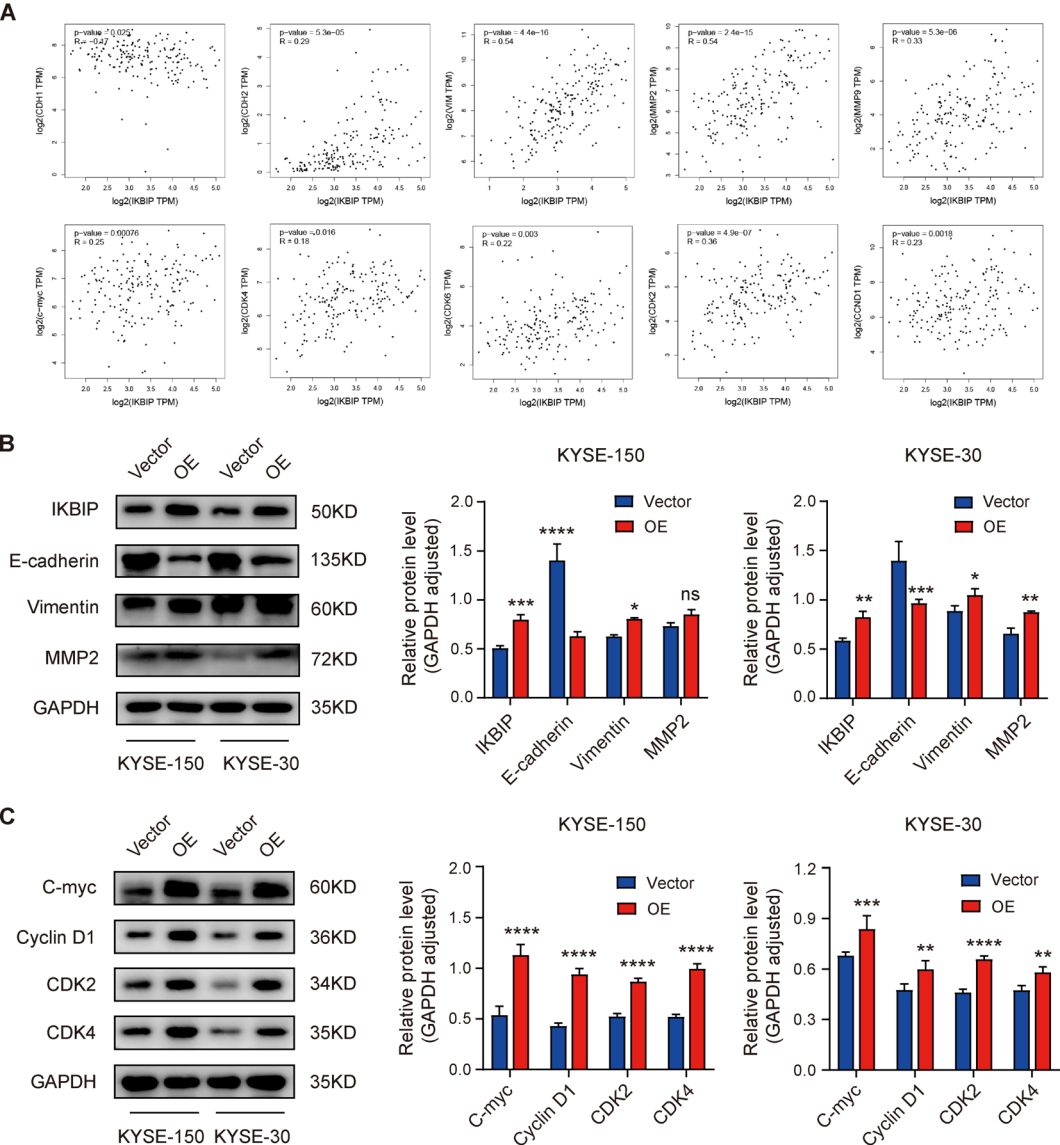
Effects of IKBIP overexpression on cell proliferation- and migration-related proteins

Since activation of the AKT signaling pathway has been reported to promote epithelial mesenchymal transformation (EMT) and the transition of tumor cells from the G1 phase to the S phase, we compared the expression patterns of IKBIP with those of cell proliferation-related proteins, EMT-related proteins and migration-related proteins in ESCA samples using the correlation analysis module in the GEPIA2 database (Fig. 6A). Pearson correlation analysis revealed that IKBIP was positively correlated with N-cadherin (*R* = 0.29, *p* < 0.0001), Vimentin (*R* = 0.54, *p* < 0.0001), MMP2 (*R* = 0.54, *p* < 0.0001), MMP9 (*R* = 0.33, *p* < 0.0001), C-myc (*R* = 0.25, *p* = 0.00076), CDK4 (*R* = 0.18, *p* = 0.016), CDK6 (*R* = 0.22, *p* = 0.003), CDK2 (*R* = 0.36, *p* < 0.0001) and Cyclin D1 (*R* = 0.23, *p* = 0.0018), and negatively correlated with E-cadherin (*R*=-0.47, *p* = 0.025). Afterwards, we verified the outcomes of the above correlation analysis through Western blotting. The expression levels of IKBIP, E-cadherin, Vimentin and MMP2 in ESCC cells with IKBIP overexpression were significantly greater than those in control cells (Fig. 6B). The expression levels of C-myc, Cyclin D1, CDK2 and CDK4 were upregulated in ESCC cells overexpressing IKBIP (Fig. 6C). The above results indicated that IKBIP promoted the process of EMT and enhanced the migration and invasion of ESCC cells. After overexpression of IKBIP, the expression of cell cycle-related proteins further increased, which was consistent with the results of cell cycle detection by flow cytometry.

(A) Expression patterns of IKBIP and cell cycle-related proteins, EMT-related genes and migration-related genes in ESCA samples were visualized through the correlation analysis module in the GEPIA2 database. (B) Western blotting was performed to detect the protein expression of IKBIP, E-cadherin, Vimentin and MMP2 in KYSE-150 and KYSE-30 cells after IKBIP overexpression. (C) The expression of the proliferation-associated proteins C-myc, Cyclin D1, CDK2, and CDK4 was detected by Western blotting. NC, sh-1 or sh-2: KYSE-150 and KYSE-30 cells infected with the pLV-NC (NC), pLV-IKBIP-sh-1 (sh-1) or pLV-IKBIP-sh-2 (sh-2) lentiviruses. Vector or OE: KYSE-150 and KYSE-30 cells infected with the pCDH-Vector (Vector) or pCDH-IKBIP (OE) lentivirus. *p < 0.05, **p < 0.01, ***p < 0.001, ****p < 0.0001. ns: *p* > 0.05.

### Inhibiting the AKT signaling pathway suppressed IKBIP-induced ESCC cell proliferation and migration

**Fig. 7:**
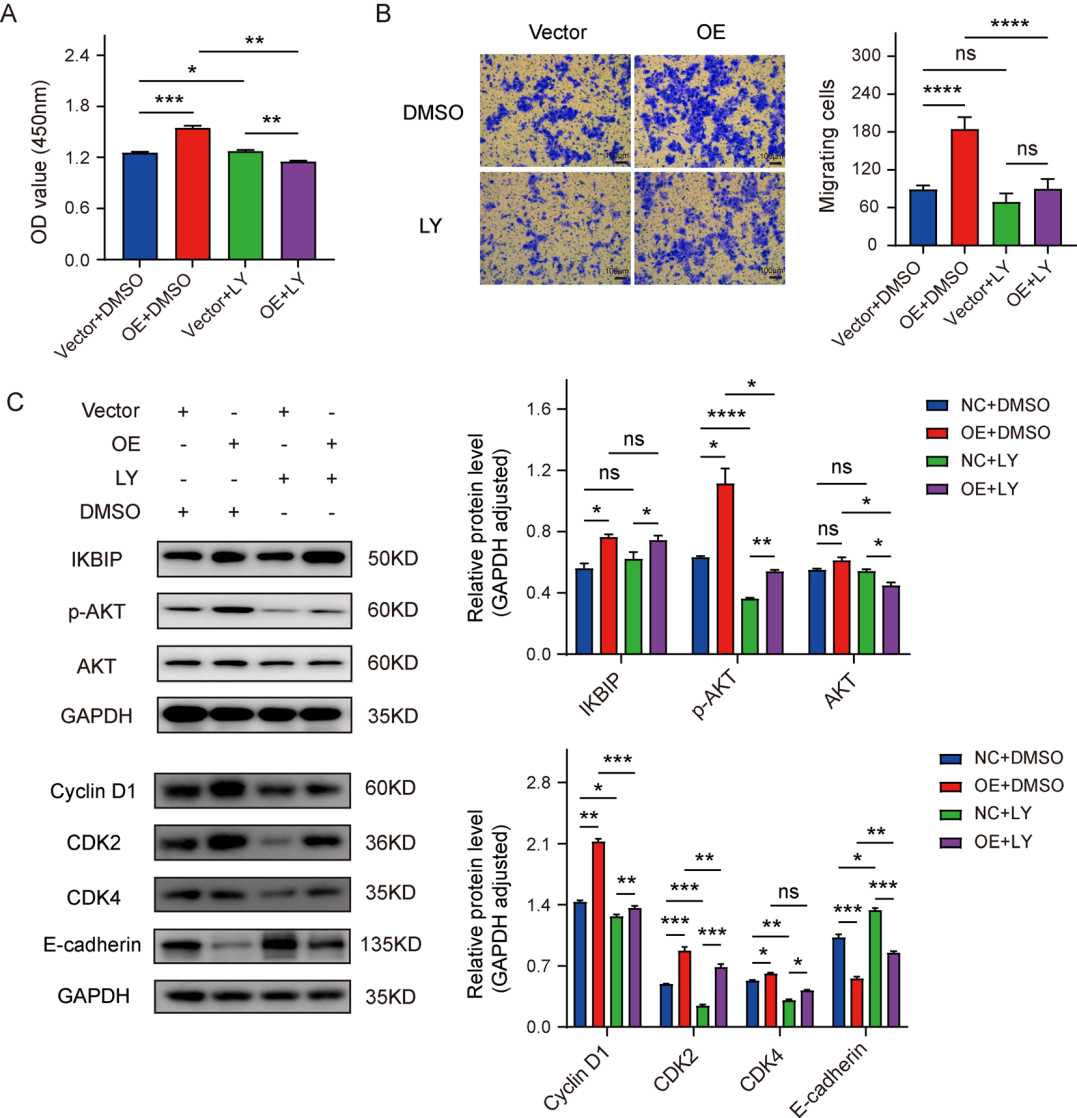
Inhibitory effects of the AKT pathway on ESCC cells overexpressing IKBIP

To confirm that the tumor-promoting effect of IKBIP was achieved by the activation of the AKT pathway, we used LY294002 (10 µg/mL), a specific inhibitor of the PI3K/AKT signaling pathway, to treat KYSE-150 cells with IKBIP overexpression (OE) and their matched control cells (Vector). We found that after adding LY294002, the proliferation (Fig. 7A) and migration (Fig. 7B) abilities of IKBIP-overexpressing KYSE-150 cells were significantly inhibited compared with the OE group treated with the same volume of DMSO. Western blot results showed that the expression of p-AKT, Cyclin D1, CDK2 and CDK4 was significantly downregulated, while E-cadherin was up-regulated after the addition of LY294002 (Fig. 7C). Notably, we did not observe significant differences in the expression of IKBIP between cells treated with LY294002 and cells not treated with LY294002, suggesting that IKBIP may be located upstream of the AKT signaling pathway.

The KYSE-150 cells overexpressing IKBIP and paired control cells were treated with 10 µg/mL of LY294002 or DMSO for 48 h, and the results of CCK-8 (A) and Transwell assays (B) were shown. The expression levels of IKBIP, p-AKT, AKT, Cyclin D1, CDK2, CDK4 and E-cadherin were detected in KYSE-150 cells overexpressing treated with LY294002 (C). LY: 10 µg/mL of LY294002. DMSO: solvent control (same volume of LY294002). *p < 0.05, **p < 0.01, ***p < 0.001, ****p < 0.0001. ns: *p* > 0.05.

### IKBIP promoted ESCC tumor growth in xenograft mice

**Fig. 8:**
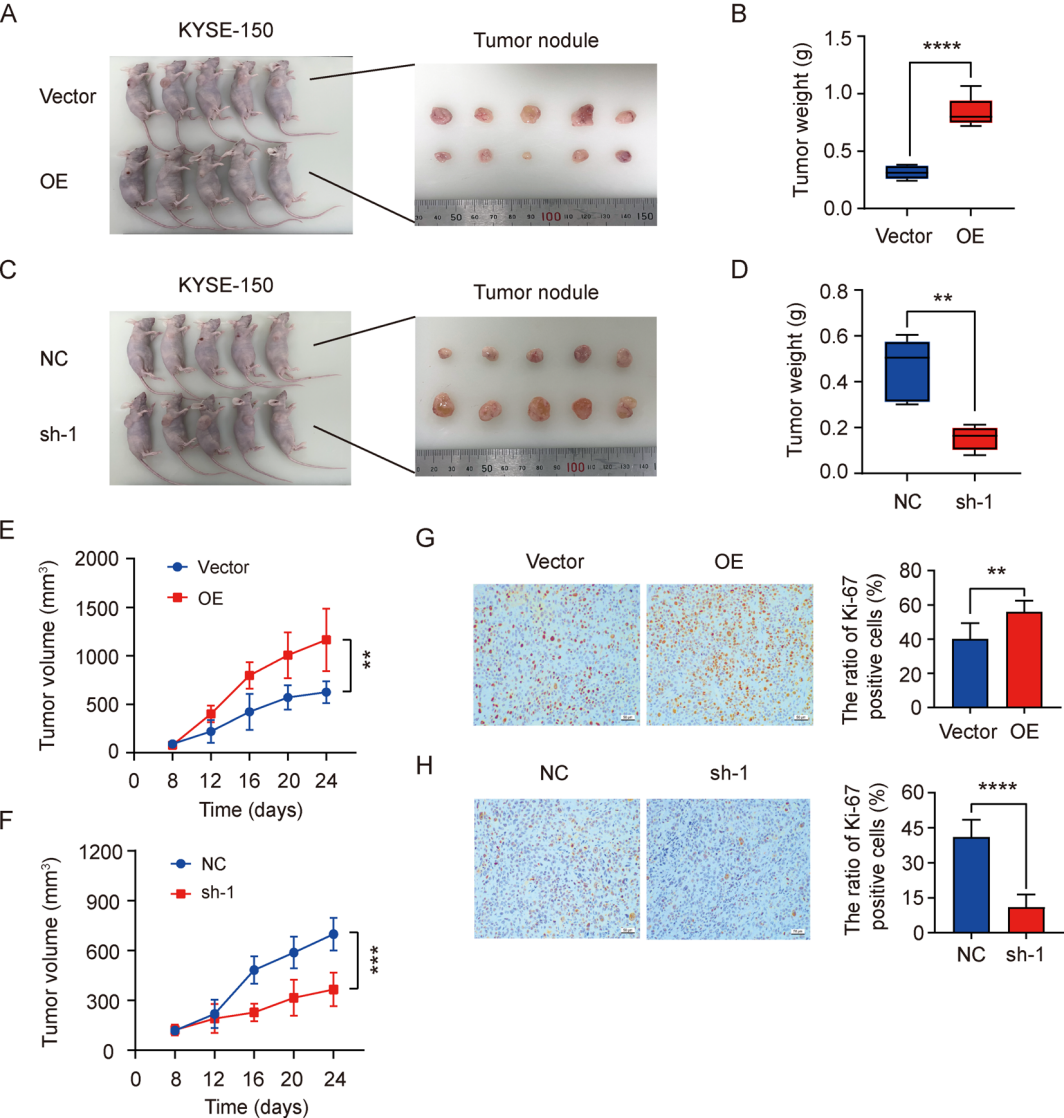
Effects of IKBIP on tumor growth in xenograft mice

To further verify whether IKBIP promotes ESCC development in vivo, we constructed tumor cell-derived xenograft models (NC, sh-1, Vector and OE) by subcutaneously injecting 1 × 10^6^ KYSE-150 cells transfected with pLV-NC, pLV-IKBIP-sh1, pCDH-Vector or pCDH-IKBIP into the right axilla of nude mice. After monitoring the tumor size for 24 days, all mice were euthanized, and the tumor nodules were removed and weighed. During this period, we did not observe tumor metastasis in tumor-bearing mice. In vivo experiments revealed larger tumor sizes (Fig. 8A**)** and greater tumor weights (Fig. 8B) in the IKBIP-overexpressing group than in the vector group. In contrast, tumor growth in the IKBIP-knockdown group was significantly inhibited compared to that in the matched control group. (Figure 8C and D). The tumor growth curve showed a significantly faster tumor growth trend in mice after IKBIP overexpression (Fig. 8E), while the IKBIP-knockdown group had a slower tumor growth trend (Fig. 8F). Subsequently, the stripped tumor tissues were subjected to IHC staining for Ki-67 expression to assess tumor growth. The Ki-67 expression in the tumor tissues of the IKBIP-overexpressing group was significantly greater than that in the tumor tissues of the vector group (Fig. 8G), whereas Ki-67 expression in the IKBIP-knockdown group was significantly greater than that in the NC group (Fig. 8H). In conclusion, we found that IKBIP overexpression significantly accelerated ESCC progression both in vitro and in vivo. Inhibition of IKBIP may be a new strategy for targeted therapy of ESCC.

A and B. The tumor nodules were isolated from the IKBIP-overexpressing group and the corresponding control group (A), and the tumor nodules were weighed (B). C and D. The tumor nodules were isolated (C) and weighed (D) from the IKBIP-knockdown group and paired control group mice. E and F. The tumor growth curves were plotted according to the tumor volume of mice in the IKBIP-overexpressing group (E) or IKBIP knockdown group (F). G and H. Representative images of Ki-67 expression in tumor samples from the IKBIP-overexpressing (G) group or IKBIP-knockdown group (H). Scale bar: 100 μm. NC or sh-1: The nude mice were subcutaneously injected with 1 × 10^6^ KYSE-150 cells transfected with pLV-NC (NC) or pLV-IKBIP-sh-1 (sh-1). Vector or OE: Nude mice were subcutaneously injected with 1 × 10^6^ KYSE-150 cells transfected with pCDH-Vector (Vector) or pCDH-IKBIP (OE). *p < 0.05, **p < 0.01, ***p < 0.001, ****p < 0.0001. ns: *p* > 0.05.

## Discussion

In recent years, an increasing number of studies have shown that IKBIP can serve as a predictive biomarker for many cancers, such as glioma [12, 13], renal cell carcinoma [14] and gastrointestinal cancer [26]. However, whether IKBIP can be used as a biomarker for ESCC is unknown. For this reason, we analyzed the IKBIP mRNA expression differences between ESCC tumor tissues and normal tissues through multiple bioinformatics databases, and found that IKBIP expression was significantly up-regulated in ESCC tissues. In tumor cells, DNA methylation usually leads to a decrease in tumor suppressor expression, that is, the inhibition of gene transcription activity [27]. We found the DNA methylation level of IKBIP was lower in ESCC tissues than in normal tissues. To explore the protein expression of IKBIP in ESCC tissues, we performed IHC staining on 126 ESCC tissues and 108 adjacent non-tumor tissues. Compared with adjacent normal tissues, IKBIP protein expression levels were significantly increased in ESCC tissues. According to the IHC score, we divided the 126 ESCC patients into high-IKBIP expression group and low-IKBIP expression group. Kaplan-Meier survival analysis showed that IKBIP expression was related to poor prognosis of ESCC patients (*p* = 0.0149). Since the choice of clinical treatment options for ESCC largely depends on patients’ clinicopathological parameters, we further analyzed the relationship between IKBIP expression and clinicopathological parameters in ESCC. IKBIP expression was significantly associated with tumor length (*p* = 0.028), N stage (*p* < 0.001), and pTNM stage (*p* < 0.001). By searching the UALCAN database, we found the expression of IKBIP increased with tumor stage, N stage and tumor differentiation grade, indicating that IKBIP may become a new biomarker for ESCC prognosis. Currently, biomarkers available for clinical diagnosis of ESCC are still limited, including squamous cell carcinoma antigen, carcinoembryonic antigen and carbohydrate antigen 19 − 9 [28], and are not highly specific. Combining these commonly used biomarkers with IKBIP may help improve the diagnosis and prognosis of ESCC.

The development of ESCC usually involves multiple malignant characteristics, such as excessive proliferation, enhanced metastasis, and enhanced drug resistance of tumor cells [29, 30]. Based on the clinical significance of IKBIP in ESCC, we further studied the biological function of IKBIP in ESCC progression both in vitro and in vivo. We used a lentiviral genetic engineering vector to disrupt or overexpress IKBIP in both KYSE-30 cells with naturally low IKBIP expression and KYSE-150 cells with naturally high IKBIP expression. In vitro functional experiments showed that IKBIP silencing significantly inhibited the proliferation, migration and survival of ESCC cells. The flow cytometry results showed that IBKIP silencing induced ESCC cell apoptosis and significantly increased the proportion of tumor cells arrested in the G1/S phase. In contrast, overexpression of IKBIP enhanced the malignant behavior of KYSE-150 and KYSE-30 cells. An in vivo study indicated that IKBIP overexpression promoted tumorigenesis and tumor growth in xenograft models established with lentivirus-transfected ESCC cells. IHC analysis of isolated tumor tissues revealed that the expression of Ki-67 in the tumor tissues of the IKBIP-overexpressing group was significantly greater than that in the corresponding control group, indicating that IKBIP plays a tumor-promoting role in ESCC progression in vivo. It should be emphasized that the role of IKBIP in different tumors may vary depending on the tumor tissue type, tumor microenvironment, and position in the gene regulatory network [31]. At present, the functional exploration of IKBIP is limited to glioblastoma and has not been studied in detail in other cancers. Our results revealed that IKBIP functions as a tumor promoter in ESCC and provides a new therapeutic target for ESCC.

The PI3K/AKT pathway is one of cellular transduction pathways that responds to external growth factor signals and plays a key role in cell growth, metabolism and survival [32]. Abnormal activation of AKT signaling may be induced by a variety of risk factors, including overexpression of carcinogenic factors, loss of tumor suppressor factors, overactivation of growth factor receptors and inflammatory stimulation [33]. These risk factors interact and influence each other, contributing to the formation and development of tumors. In breast cancer, phosphorylated AKT promoted tumor cell proliferation and cell cycle progression by activating the mTOR complex [34]. It has been reported that activated AKT further phosphorylates the GSK-3β protein, inhibits the expression of E-cadherin, and promotes the process of EMT, which results in tumor migration and invasion [35]. In this study, we showed that IKBIP was related to the activation of AKT signaling pathway. IKBIP overexpression increased the expression of cell cycle-related proteins (C-myc, Cyclin D1, CDK2 and CDK4), EMT-related proteins (E-cadherin and vimentin) and the cell migration-related protein MMP2, thereby promoting the proliferation and migration of ESCC cells. To verify the role of the AKT signaling pathway in the promotion of ESCC development by IKBIP, we inhibited AKT signaling with LY294002. We found that the proliferation and migration abilities of IKBIP-overexpressing KYSE-150 cells treated with LY294002 were significantly reduced compared with those of OE cells not treated with LY294002 but were still greater than those of control cells treated with LY294002. In addition, inhibition of the AKT pathway did not change the self-expression of IKBIP in KYSE-150 cells, suggesting that IKBIP may be located upstream of the AKT signaling pathway. Based on these results, we speculate that PI3K/AKT signaling inhibitors may be effective at inhibiting tumor development in ESCC patients with high IKBIP expression but may be ineffective at preventing tumor development in ESCC patients with low IKBIP expression. Our results may provide new ideas for clinical targeted therapy of ESCC.

It should be noted that we did not answer how IKBIP promoted the activation of the AKT signaling pathway. In addition, the small number of ESCC samples involved in this study may limit our findings, and further in-depth studies based on larger sample sizes, more clinical factors, as well as multi-center collaboration are needed to dissect the exact clinical significance of IKBIP in ESCC.

## Conclusion

IKBIP expression was significantly increased in ESCC tissues and was closely associated with the prognosis of ESCC patients. Knockdown of IKBIP significantly inhibited the proliferation, survival, and migration of ESCC cells and inhibited tumor growth in xenograft nude mice. In contrast, IKBIP overexpression promoted the development of ESCC both in vitro and in vivo, which may be related to the activation of the AKT signaling pathway by IKBIP. In summary, we revealed the potential of IKBIP as a biomarker for predicting ESCC and the role of IKBIP in promoting tumor development in ESCC. IKBIP may be a new target for the clinical treatment of ESCC.

### Electronic supplementary material

Below is the link to the electronic supplementary material.

Supplementary Material 1

## Data Availability

The datasets supporting the conclusions of this article are available in the GEO repository, [GSE199967 https://www.ncbi.nlm.nih.gov/geo/query/acc.cgi? acc=GSE199967; GSE164157 https://www.ncbi.nlm.nih.gov/geo/query/acc.cgi]. Public databases used in this article are GEPIA2 [http://gepia2.cancer-pku.cn/#index] and UALCAN [https://ualcan.path.uab.edu/]. The RNA-seq data that support the findings of this study within the supplementary information files. Experimental data and materials for this study are available from the corresponding author upon reasonable request.
